# Reducing Metabolic Syndrome through a Group Educational Intervention Program in Adults with Obesity: IGOBE Program

**DOI:** 10.3390/nu14051066

**Published:** 2022-03-03

**Authors:** Cristina Tejera, Cristina Porca, Gemma Rodriguez-Carnero, Paula Andújar, Felipe F. Casanueva, Diego Bellido, Ana B. Crujeiras

**Affiliations:** 1 Division of Endocrinology and Nutrition, Complejo Hospitalario Universitario de Ferrol (CHUF/ SERGAS), 15405 Ferrol, Spain; cris88_pf@hotmail.com (C.P.); diegobellido@gmail.com (D.B.); 2 Epigenomics in Endocrinology and Nutrition Group, Instituto de Investigación Sanitaria de Santiago (IDIS), Complejo Hospitalario Univeristario de Santiago de Compostela (CHUS/SERGAS), 15706 Santiago de Compostela, Spain; mrcarnero@alumni.unav.es (G.R.-C.); anabelencrujeiras@hotmail.com (A.B.C.); 3 Division of Endocrinology and Nutrition, Complejo Hospitalario Universitario de Santiago de Compostela (CHUS/SERGAS), 15706 Santiago de Compostela, Spain; paula.andujar.plata@sergas.es; 4 Molecular Endocrinology Group, Instituto de Investigacion Sanitaria de Santiago (IDIS), Complejo Hospitalario Universitario de Santiago de Compostela (CHUS/SERGAS), 15706 Santiago de Compostela, Spain; felipe.casanueva@usc.es; 5 Centro de Investigacion Biomedica en Red Fisiopatologia de la Obesidad y Nutricion (CIBERobn), 28029 Madrid, Spain

**Keywords:** obesity, metabolic syndrome, weight loss, behavioral group

## Abstract

Metabolic syndrome (MetS) increases the risk of cardiovascular disease, type 2 diabetes mellitus, and cancer. Despite the higher prevalence of MetS in obese adults, little is known about the effectiveness of intensive and group interventions in improving MetS prevalence. This study aimed to investigate the effectiveness of an intensive lifestyle program in reducing the prevalence of MetS in adults with obesity. Patients with obesity (*n* = 456, 48.8 ± 12.8 years, 18.5% male) were randomized in two groups as indicated in a prospective interventional real-life study: a control group (CG), in which patients received usual care, and an interventional group (IG), in which the patients participate in a healthy lifestyle habits program in six weekly sessions, IGOBE program. Anthropometric, body composition, medications, and MetS features data were analyzed in both groups at the pre-intervention and post-intervention stages using a completer’s analysis. At 12 months of follow-up, the IG showed a relative reduction of 13.4% in the prevalence of MetS from baseline, while the CG showed a reduction of 2.1% (*p* < 0.001). A significant reduction was also observed in four of five MetS features. In this trial, implementation of the IGOBE program resulted in a significant reduction in MetS prevalence and better control of MetS features compared with the standard of care.

## 1. Introduction

Obesity is a major public health problem with an exponentially increasing incidence [1] and affects 21.6% of adults in Spain [2]. The World Health Organization defines obesity as excessive fat accumulation that may impair health and proposes the use of a cut-off body mass index (BMI) value of greater than or equal to 30 kg/m^2^. The same cut-off value is used by the Centers for Disease Control and Prevention, which divides obesity into three categories: class 1 for BMI of 30 to <35 kg/m^2^, class 2 for BMI of 35 to <40 kg/m^2^, and class 3 for BMI of 40 kg/m^2^ or higher [3]. The causes of obesity are complex and mainly result in an energy imbalance that promotes fat storage. The main complications associated with obesity include metabolic syndrome, cardiovascular disease, musculoskeletal disorders, and some types of cancer. Obesity remains a challenge to the healthcare systems owing to its associated complications, healthcare costs due to related features or medical consultations between others, and social costs. One of the most relevant complications associated with obesity is metabolic syndrome (MetS) in terms of frequency and associated complications.

MetS is a cluster of metabolic alterations that includes abdominal obesity, dyslipidemia, high blood pressure, and hyperglycemia [4]. Insulin resistance is the primary cause of most of the metabolic alterations, but other mechanisms have emerged that can promote the development of MetS and its complications, such as genetic predisposition; increases in the levels of angiotensinogen, resistin, or leptin; and environmental factors [5]. MetS is common among patients with abdominal obesity and increases the risk of cardiovascular disease and type 2 diabetes. MetS also increases the risk of chronic kidney disease, fatty liver disease, and all-cause mortality [6]. Recent studies have evaluated the relationship between MetS, non-alcoholic fatty liver disease, and colonic diverticulosis and reported the role of dysbiosis in the development of MetS and its complications [7]. Moreover, MetS is a major comorbidity in approximately 20% of coronavirus disease 2019 patients, and it is associated with increased risk of short-term mortality [8].

Various diagnostic criteria have been proposed to define MetS. Recently, MetS is defined based on the criteria proposed by the International Diabetes Federation, National Heart, Lung, and Blood Institute, American Heart Association, World Heart Federation, International Atherosclerosis Society, and International Association for the Study of Obesity, commonly known as “harmonized classification” [9]. The criteria include increased waist circumference (population specific), triglyceride levels of ≥150 mg/dL or drug treatment, high-density lipoprotein (HDL) levels of <40 mg/L in men or <50 mg/dL in women or drug treatment, a systolic blood pressure of ≥130 mmHg or a diastolic blood pressure of ≥85 mmHg or drug treatment, and a fasting glucose of >100 mg/dL or drug treatment. A person who meets three out of the five criteria is diagnosed with MetS.

Similar to obesity, the first-line therapy to prevent and treat MetS is lifestyle modification, such as consumption of a healthy diet and performance of physical activities [10]. The Diabetes Prevention Program (DPP) reported a reduction of 41% in the incidence of MetS in the lifestyle modification group and 17% in the metformin group compared with that in the placebo group for participants with impaired glucose tolerance but without diabetes [11]. Results of the PREDIMED randomized trial conducted showed that the MetS prevalence rates reduced by 6.7%, 13.7%, and 2.0% after the consumption of Mediterranean diet supplemented with virgin olive oil, nuts, and low-fat diet, respectively, within 1 year in an older population at higher risk for cardiovascular disease [12]. In the Look-AHEAD study, after 1 year of an intensive lifestyle intervention in type 2 diabetes patients, the prevalence of MetS was reduced by 14.7% in this group [13].

In the endocrinology units, the treatment and follow-up of obese patients is a significant workload in terms of time and resources. Commonly, obese patients attend individual, in-person clinical visits with a limited time, have a lack of personalized therapy, are living in an environment with high prevalence of obesity, and have comorbidities. Group visits to support behavior change among obese individuals are an effective alternative to individual visits to promote changes in unhealthy diet and increase the level of physical exercise [14,15,16]. Moreover, group visits for persons with obesity provide opportunities for peer support.

The Group Intervention in OBEsity (IGOBE) is a structured program of nutrition education for obese patients based on group care and promotes the consumption of a healthy diet, performance of prescribed exercises, and provision of behavioral support in a clinical real-life setting of adults with obesity. Data regarding the changes in anthropometric parameters, body composition, and modification of lifestyle habits have been reported in previous studies [17]. At 12 months after the initiation of the IGOBE program, the intervention group (IG) achieved greater weight loss (−7.11% of the initial weight) than the control group (CG), accompanied by a reduction in fat mass, specifically visceral fat. The IG showed better adherence to healthy dietary patterns. In fact, the IGOBE program was proposed as a new and effective therapeutic approach to be implemented in real-life practice for obesity treatment [17]. However, whether the beneficial effect induced by IGOBE program on body weight, body composition, and dietary habits is also translated in an improvement in obesity-related comorbidities are still an open question.

This study aimed to evaluate the effectiveness of the IGOBE program conducted within 1 year compared with that of standard therapy for weight loss by assessing the changes in the prevalence of MetS and analyzing the modifications in each component of MetS.

## 2. Materials and Methods

### 2.1. Study Design

IGOBE is a nutritional intervention study that is open, controlled, randomized, prospective and comparative, conducted with two parallel groups for 1 year and carried out in a single center (University Hospital of Ferrol, A Coruña, Spain) [17]. The study was performed in 2013–2014.

### 2.2. Participant Selection and Recruitment

Medical doctors from the Division of Endocrinology and Nutrition of the University Hospital of Ferrol assessed the potential participants for eligibility. Men and women, individuals aged ≥18 years, individuals who were obese (body mass index of ≥30 kg/m^2^), individuals motivated to maintain healthy lifestyle habits, and individuals who attended scheduled meetings and control visits were eligible for this study. Participants who had obesity induced by endocrine problems, were diagnosed with mental illness or other health problems that could alter their response to treatment, with drug-abuse problems or consumed alcohol, use weight loss drugs, planned to get pregnant or were pregnant during the study period, previously underwent weight-loss surgery, had special dietary restrictions, and acquired an HIV infection were excluded. The criteria for withdrawal from the study were inability to attend the scheduled sessions, decision by the patient, and pregnancy.

Potentially eligible candidates were informed by a specialist in endocrinology and nutrition during their visit to the unit. The candidates were screened based on the inclusion and exclusion criteria. Afterward, patients were offered the opportunity to participate in the study, and informed consent was obtained. The study protocol was made in accordance with the Declaration of Helsinki and was approved by the Autonomic Committee of Clinical Research Ethics of Galicia, Spain (registry 2013/368).

### 2.3. Randomization and Study Protocol

Prior to randomization, an initial evaluation was performed by a specialist in endocrinology and nutrition, which involved the collection of medical history records, anthropometric measurements, body composition, and habit questionnaire parameters. This global evaluation was performed at three visits: baseline, 6 months, and 12 months. Patients were prescribed with a balanced hypocaloric diet in order to achieve a 500–1000 kcal/day reduction from the habitual intake. This recommendation is based on the guidelines of the Spanish Society for the Study of Obesity (SEEDO) 2007 [18], the American Dietetic Guidelines 2010, the Consensus FESNAD SEEDO [19] 2012, the American College of Cardiology/American Heart Association Task Force on Practice Guidelines, and the Obesity Society Guideline for the Management of Overweight and Obesity in Adults 2014 [20].

**Figure 1:**
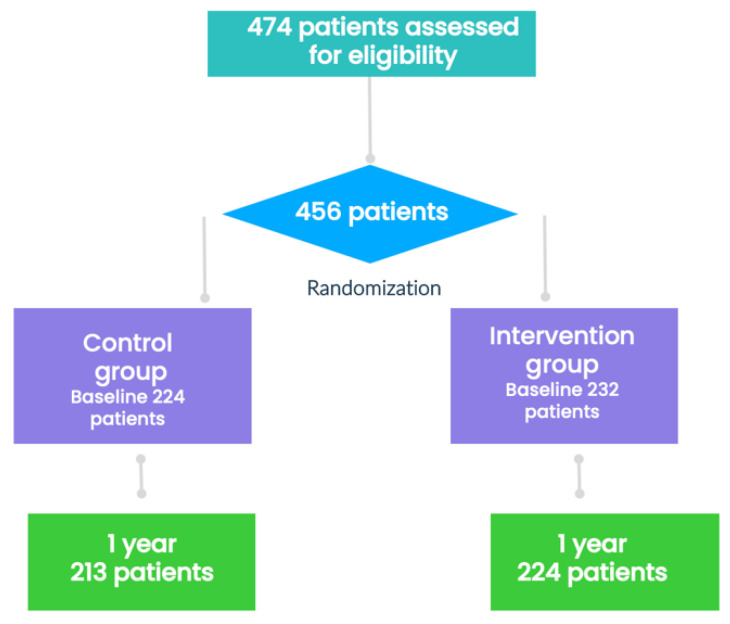
Flowchart of the participants in the study.

After the basal visit and obtaining the informed consent, the candidates were randomized in a 1:1 ratio to either the IG or CG using the Epidat^®^ program, a computer-generated random-number internet-based system. The IG and CG differed according to training, intensity, reinforcement, and social support, following the protocol. See Figure 1.

#### 2.3.1. Control Group

Participants allocated to the CG received the standard of care for obesity while admitted in the hospital; their clinical treatment was revised by an endocrinologist and endocrinology nurse at 6 and 12 months after the baseline visit, with a mandatory visit at 12 months. During this visit, the patients were encouraged to change their unhealthy lifestyle, adhere to the prescribed diet, provide their medical records, and ensure weight and body composition control. In the usual practice, after basal evaluation, the doctors and nurses provided a written prescription of the recommended diet and exercise. The patients returned for body weight control and body composition control at 6 and 12 months after the baseline visit. No other contact or considerations were made during follow-up.

#### 2.3.2. Intervention Group

The participants allocated to the IG followed a structured program of habit change and exercise. This program included a baseline visit, an intensive program of six weekly 1 h sessions, and two follow-up visits at 6 and 12 months. If the entire session had an attendance rate of 80%, the program was considered successful.

In the IG, the nutritionists and nurses conducted the six weekly 1 h sessions with 15 patients per group using the active-participation method, encouraging communication and interactive learning (see IGOBE program previous publication for more details [17]). The topics discussed during the sessions were preparation of menus, healthy recipes, preparation of healthy eating plans, knowledge about labeling, methods of managing emotional hunger, and registration of weekly activities. Workout involved the performance for 360 min of weekly physical activity or 10,000 steps/day and included strength work. In all sessions, 15 min were allotted for performing exercise. During the session, behavioral therapy was performed, and discussion groups was conducted to maximize the “halo effect” of peer interaction on eating habits, exercise, and healthy lifestyle. The weekly sessions aimed at providing the patients with knowledge and tools to promote healthy habits and resources in order to encourage patients to maintain a healthy lifestyle. In this session, false beliefs were discussed, and real-life experiences were provided as examples on how the theory is applied to day-to-day life.

In addition, the IG was provided with a social support system through the establishment of an e-mail support and a website containing information and healthy recipes (www.foroactua.com, accessed on 21 February 2022), incorporated as part of the program; received e-mails to reinforce the message; and underwent continuous training by following the information posted on the website. Participants were encouraged to register in paper or mobile phone apps the food they consumed, training they received, and their feelings to track their improvements and to provide them with tips and strategies to help them achieve success.

After finishing the six sessions, the participants attended two more reinforced sessions at 6 and 12 months after the beginning of the program.

### 2.4. Measurements

In this study, measurements were taken from baseline and during the 12-month group-based sessions. Trained research staff collected the measurements. Demographic information, medications, and medical history were obtained from the patient using the digital clinical history of our health care system, IANUS^®^. Data related to the medication used for hypertension, diabetes mellitus, elevated triglycerides, and elevated cholesterol levels were also collected. Habitual intake was documented using a food registration record and photographic dietary record reported by the patients.

Body weight, height, waist circumference, and body composition were measured with the patient on bare foot and in light clothing after an overnight fasting of 8–10 h. Body weight and height were measured twice. Height was measured using a calibrated stadiometer (Seca 220^®^, Medical Resources, EPI Inc., Lewis Center, OH, USA), while weight and body composition were measured using a bioelectrical impedanciometer (In Body 720^®^, Biospace Inc., Tokyo, Japan). The visceral area values were calculated in cm^2^ using multifrequency bioelectrical impedance analysis. Waist circumference was measured with a non-elastic metric tape between the lower costal margins and iliac crest.

An automated blood pressure machine (Omron Digital Blood Pressure Monitor M7^®^, Matusaka Co. Ltd., Kubocho Matsuzaka, Japan) was used to measure the systolic and diastolic blood pressure levels twice in a quiet area.

After an overnight fast, the blood samples were collected at baseline and 12 months. The serum and plasma samples were collected and analyzed in the laboratory at our center. The serum glucose, triglyceride, total cholesterol, and HDL cholesterol levels were measured using standard enzymatic methods, while the low-density lipoprotein (LDL) cholesterol was calculated using the Friedewald formula.

MetS was defined using the criteria provided by the International Diabetes Federation, National Heart, Lung, and Blood Institute, American Heart Association, World Heart Federation, International Atherosclerosis Society, and International Association for the Study of Obesity [9]. This definition includes increased waist circumference, which is population specific; for this proposal, we used the cutoff values for the population that were reported previously [21]: 97 cm in men and 88 cm in women. Specifically, patients with three or more of the following five risk factors were diagnosed with MetS: (1) waist circumference measurements of ≥97 cm in men and ≥88 cm in women, (2) triglyceride levels of ≥150 mg/dL or drug treatment, (3) an HDL level of <40 mg/L in men or an HDL level of <50 mg/dL in women or drug treatment, (4) a systolic blood pressure of ≥130 mmHg or a diastolic blood pressure of ≥85 mm Hg or drug treatment, and (5) a fasting glucose level of >100 mg/dL or drug treatment.

### 2.5. Statistical Analyses

The sample size was calculated as previously reported [17]. Data were processed using the SPSS statistical package (SPSS for Windows version 24, SPSS Inc., Chicago, IL, USA). Continuous variables were expressed as mean ± SD if the distribution was normal or as median and range if the distribution was non-normal. Qualitative variables were described as absolute and relative frequencies (percentages). The data were collected in tables and presented in graphs for each type of variable (bar diagrams for qualitative variables and frequency histograms for quantitative variables). To study the association between qualitative variables, the chi-square test was used with Yates correction and Fisher’s exact test when required. For quantitative variables, the Kolmogorov–Smirnov test was used to determine the normality of the distributions. The relationships between quantitative variables were analyzed using Pearson’s correlation tests (under parametric conditions) or Spearman’s correlation tests (under non-parametric conditions). Parametric or non-parametric statistical tests required by the application conditions were used to study the differences between independent means. A repeated-measures analysis of covariance was used to assess the differences over time in anthropometric parameters, body composition, and MetS status between groups adjusted for age and sex. The level of significance was set at *p* ≤ 0.05.

## 3. Results

### 3.1. Baseline Characteristics

Of the 474 patients assessed for eligibility, 456 were randomly assigned to either the CG or IG. After discounting withdrawals, the MetS status at 1 year was assessed in 437 participants (213 in the CG and 224 in the IG). Of the 437 patients, 81 (18.5%) were men and 356 (81.5%) women. The participants were aged between 18 and 77 years (average: 48.8 ± 12.8 years, median: 49 years). The groups were well balanced in terms of demographic characteristics, CV risk factors, MetS prevalence, and medication use except for the age, which was slightly higher in IG than in CG (50.2 ± 12.1 years vs. 47.3 ± 13.4 years, *p* = 0.021). The percentage of patients with a total cholesterol level of 200 mg/dL was higher in CG than IG (46.2% vs. 36.9%, *p* = 0.028). A total of 267 patients (61.1%) met the harmonized criteria for diagnosing MetS, and the distribution among groups was similar. The baseline characteristics of the study participants are presented in Table 1. Data regarding total daily intake have been published previously [17].

### 3.2. Changes in Body Weight, Body Composition, Waist Circumference, and Blood Pressure

In the CG, the mean ± SD blood pressure levels were 137 ± 17 mmHg and 82 ± 11 mmHg, respectively. In the IG, the mean ± SD of blood pressure levels was as follows: systolic blood pressure level of 138 ± 17 mm Hg and diastolic blood pressure level of 82 ± 10 mm Hg, without statistical differences at baseline for both groups. At 12 months, the blood pressure levels did not differ between the CG and IG: systolic blood pressure level of 135 ± 18 mmHg and diastolic blood pressure level of 79 ± 13 mmHg vs. systolic blood pressure level of 134 ± 17 mmHg and diastolic blood pressure level of 80 ± 11 mmHg (*p* = 0.597 and *p* = 0.345, respectively).

Data on changes in body weight, body composition, and waist circumference are shown in Table 2.

### 3.3. Metabolic Syndrome 

**Figure 2:**
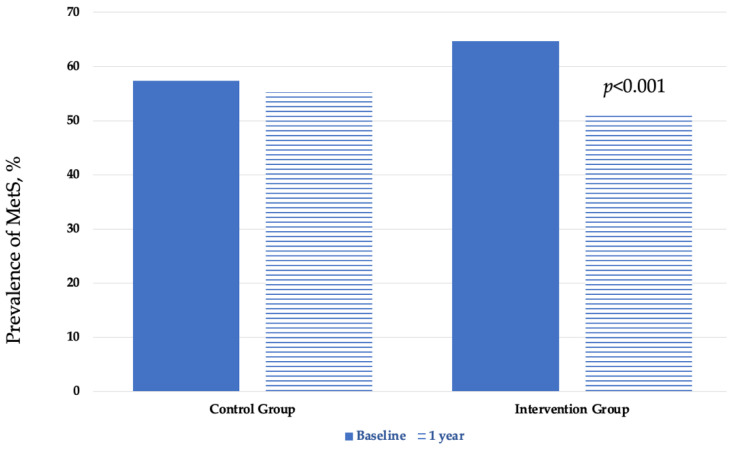
Baseline and 1-year prevalence of the metabolic syndrome by group.

A total of 267 patients (61.1%) met the harmonized criteria for diagnosing MetS, and the distribution among groups was similar (see Table 1). MetS was more frequent in men than in women (*p* < 0.0001) but not at the end of the study period. At the beginning of the IGOBE program, the prevalence rates of MetS were 57.3% in the CG and 64.7% in the IG (*p* = 0.542). After 1 year of follow-up, the prevalence rates of MetS were 51.3% in IG and 55.2% in CG (*p* = 0.004). The prevalence rates of MetS at 1-year assessment was reduced by 2.1% in the in CG and by 13.4% in the IG (*p* < 0.001) (see Table 3 and Figure 2).

### 3.4. Metabolic Syndrome Components

The most common MetS feature were high triglyceride levels (97.9%), abdominal obesity (88.5%), and elevated blood pressure levels (72.3%). The less common MetS features were hyperglycemia (47.3%) and low HDL levels (36.1%).

At 1 year, the IG presented a significant improvement compared to the CG in four of the five components of MetS: abdominal obesity (−41.5% vs. −33.8%), high fasting serum glucose level or use of antidiabetic drugs (−13% vs. −5.2%), low HDL levels (−4.4% vs. −3.7%), and high blood pressure or use of antihypertensive drugs (−7.9% vs. −6.5%). (see Table 3). 

### 3.5. Medications 

Antihypertensive drugs were the most common medication (40.5% in CG and 50.9% in IG), with no differences between the groups. The numbers of antihypertensive drugs taken were as follows: one medication, 22.5%/31%; two medications, 14%/19.5%; three medications, 5.5%/6%; and four medications, 1%/0.5%, respectively in CG and IG. No differences were observed at the end of the study period (see Table 4).

As regards the treatment for lower cholesterol, the rate of medication use was initially similar in both groups (*p* = 0.890), with statins being the most frequent medication (28.3% in CG and 30.8% in IG).

On the contrary, the rate of fibrate consumption was less common, that is, 4.7% in CG and 2.6% in IG. No differences were observed in the use of fibrates at baseline or at the end of the study period.

Use of hypoglycemic agents and insulin was similar in both groups at baseline: 24% and 8%, respectively. The mean number of medications consumed was 1.7 medications/patient. The mean units of insulin administered were 57 ± 43 units for the CG and 51 ± 25 units in IG. DPP-4 inhibitor use was higher in the IG group (*p* = 0.008). No significant differences were observed at 1 year (*p* = 0.475). A significant reduction in insulin dose was reported in the IG (final doses: 48 ± 33 units for CG and 34 ± 20 units for IG; *p* = 0.045).

### 3.6. Laboratory Test

The main results are presented in Table 5. No significant differences were observed in any of the analyzed parameters.

## 4. Discussion

In this trial, the implementation of the IGOBE program resulted in a significant reduction in MetS prevalence and better control of MetS features compared with the use of the standard of care. The results demonstrated a significant reduction in body weight and body fat and visceral fat. Moreover, implementation of this program resulted in better adherence to a healthy dietary pattern and change in eating habits as shown in previously published data [17]. The MetS prevalence significantly reduced by 13.4% in the IG. The current trial demonstrates that the IGOBE program based on an intensive group nutritional educational intervention, together with behavioral components and exercise prescription, is able to reduce the MetS prevalence and improves individual MetS features in a better way than a regular hospital-based therapy of obesity. This study further reinforces the suitability of IGOBE program as a model of routine clinical practice for fighting against obesity and its co-diseases [17].

In comparison with other lifestyle intervention study groups, the results were similar. The baseline prevalence of MetS was analogous to that of other studies, such as the DPP study [11], PREDIMED [12], and Look AHEAD [13]. In the DPP study [11], among people with impaired glucose tolerance, the MetS prevalence reduced by 12% in the intervention group according to ATP III criteria (The National Cholesterol Education Program’s Adult Treatment Panel III) [22]. In the PREDIMED study [12], in older adults with higher risk of cardiovascular disease, the rates of MetS prevalence according to the ATP III criteria reduced by 13.7% in the Mediterranean diet with nuts group, 6.7% in the olive oil group, and 2% in control groups after 1 year of intervention. In the Look AHEAD trial, in persons with type 2 diabetes, the MetS prevalence according to the ATP III criteria reduced by 14.7% after 1 year of intensive lifestyle intervention. Importantly, under the IGOBE program, an improvement in the percent of specific MetS components was observed, such as a reduction in percent of patients with abdominal obesity, high fasting serum glucose levels or use of antidiabetic drugs, low HDL levels and high blood pressure or use of antihypertensive drugs.

This effect was not reflected by changes in the mean levels of biochemical parameters but in reduction in body weight and body composition, particularly visceral fat mass. It suggests that the reduction in fat mass could be the first-line parameter involved in the beneficial effect observed regarding to the MetS prevalence induced by the IGOBE program. Excess adiposity uses to be associated with a dysfunctional adipose tissue, mainly visceral depot, which produce proinflammatory factors involved in the pathogenesis of obesity [23,24]. Within the MetS components, the IGOBE program is especially effective in reducing abdominal obesity, a well-known risk factor for development of MetS and its co-diseases [10]. In addition, the improvement in dietary habits induced by IGOBE program, which have been previously reported [17], could be other relevant parameter that promote the MetS reduction. Indeed, a previous study demonstrated a declining in MetS prevalence after following a Mediterranean diet, which was not associated with weight loss [12]. A more extended follow up would be needed to detect changes at biochemical parameters. No differences were reported at the end of the study regarding medications, only insulin, the need for which decreased significantly in IG, which is expected when weight and insulin resistance decreases. The novelty of IGOBE program is that a positive effect on MetS was achieved by an intensive lifestyle program focus in the obesity group program, without use of drugs. IGOBE program contributes to the management of the person with obesity in accordance with the proposal of obesity as a chronic diseased based on adiposity [25].

In terms of the lipid profile and, more specifically, in triglyceride levels, there were no significant differences between groups at the end of the study. Although cardiovascular disease risk is increased when fasting triglycerides are >1.7 mmol/L (150 mg/dL), the available evidence on the cardiovascular benefit of controlling triglyceride levels even through pharmacological measures is very weak. The ACCORD study [26] and the FIELD study [27] showed benefit exclusively in a subgroup of patients with type 2 diabetes mellitus with high triglyceride levels over 200 mg/dL and low HDL. Furthermore, the REDUCE-IT [28] study showed a reduction in cardiovascular events with the use of icosapent ethyl, whose benefit was independent of the triglyceride levels. This lack of evidence is reflected in the clinical practice guidelines where the use of drugs to lower triglyceride levels may only be considered in high-risk patients when triglycerides are >2.3 mmol/L (200 mg/dL).

It is important to highlight that a reduction in the prevalence of MetS is associated with a decrease in insulin resistance and control of well-known cardiovascular risk factors [6,10]. Insulin resistance per se increases the cardiovascular risk. In fact, a more intensive approach should be used in obese patients due to their increased risk of developing cardiovascular diseases [29]. Moreover, reduction of MetS prevalence is indirectly related to a decreased risk of other associated complications, such as chronic kidney disease, fatty liver disease, all-cause mortality, and colonic diverticulosis [6,7,9,10].

The trial was designed to be integrated into routine clinical practice and carried out by personnel forming part of the unit. No resources other than those available at the center were used. One of the main strengths of the IGOBE program is that it is a randomized trial, which confers scientific solidity to the study. During group visits, there was a high attendance rate due to the involvement of professionals and the reinforcement obtained during peer interactions in every group visit. The percentage of dropout was similar to that in other related studies [11,12,13]. In addition, the intervention provided digital support. The longitudinal design is another strength of this study; this study not only evaluated the changes in body weight and body composition but also analyzed the dietary habits and comorbidities associated with obesity. Reduction of weight and body fat are important objectives in obesity treatment; however, the ultimate goal is to promote a healthy lifestyle, avoid the development of complications associated with obesity, and reduce pre-existing complications.

This study has several limitations. First, the sample was predominantly women, as reported in similar studies [11,12,13]. Women who tend to complain about being obese are more conscious of the problems and social pressure associated with this condition, while men value their health status more positively and are less likely to consult healthcare professionals about their obesity [30]. Second, the study data on diet follow-up were auto-reported. Third, although physical activity recommendations were provided, the data were not systematically collected. Fourth, the socioeconomic status of the participants was not assessed. However, these limitations do not invalidate the results because the cohort of patients included in this study is representative of a hospital-based population.

In conclusion, the IGOBE program proved to be more effective than the usual management of obesity, driving improvements in the prevalence of MetS and of four of its five specific components. Based on these results, the advantages of weight loss and the possibility of integrating it into daily clinical practice have made the IGOBE program the standard of care for obesity in our unit. Even patients who are candidates for bariatric surgery must follow the program prior to surgery. The IGOBE program could be exportable and reproducible to other centers for obesity treatment and can be used as a tool to improve an individual’s understanding of this pathology beyond body weight control.

## 5. Conclusions

Among patients with obesity, the IGOBE program, an intensive lifestyle 12-month, multidisciplinary, and in-group intervention, was more effective than the usual management of obesity in decreasing MetS prevalence and four of five of its components. Based on these results, the IGOBE program is the standard of care for obesity in our unit.

## Figures and Tables

**Table 1:** Baseline characteristic of participants who completed the 1-year IGOBE program.

Variable	Global (*n* = 437)	Control Group (*n* = 213)	Intervention Group (*n* = 224)	*p*-Value
**General characteristics**				
Age, y, mean (SD)	48.8 ± 12.8	47.3 ± 13.4	50.2 ± 12.1	0.021
Men, *n* (%)	81 (18.5)	37 (17.4)	44 (19.6)	0.541
Current smokers, *n* (%)	82 (18.7)	44 (20.6)	38 (16.9)	0.744
Body weight, kg, mean (SD)	105.3 ± 221.5	102.6 ± 18.3	107.8 ± 23.9	0.772
BMI, kg/m^2^, mean (SD)	39.9 ± 7.0	39.2 ± 5.6	40.5 ± 7.9	0.716
Waist circumference, cm, mean (SD)	113.9 ± 15.7	111.5 ± 15.2	115.8 ± 16.0	0.863
Fat mass, kg, mean (SD)	50.2 ± 12.5	48.9 ± 11.7	51.4 ± 13.1	0.898
Fat mass, %, mean (SD)	48.0 ± 5.5	46.1 ± 5.8	48.3 ± 5.1	0.923
Visceral fat, cm^2^, mean (SD)	209.0 ± 50.6	201.8 ± 48.8	215.2 ± 51.6	0.914
Hypertension ^a^, *n* (%)	197 (45.1)	88 (41.3)	109 (48.6)	0.123
Hypercholesterolemia ^b^, *n* (%)	180 (41.1)	98 (46.2)	82 (36.9)	0.028
Hypertriglyceridemia ^c^, *n* (%)	134 (30.6)	68 (32.7)	66 (30.0)	0.132
Type 2 diabetes mellitus, *n* (%)	102 (23.3)	49 (23.0)	53 (23.7)	0.402
Metabolic syndrome ^d^, *n* (%)	267 (61.1)	122 (57.3)	145 (64.7)	0.542
**Metabolic syndrome components ^d^**				
Abdominal obesity *n* (%)	387 (88.5)	180 (84.5)	207 (92.4)	0.596
Low HDL level, *n* (%)	158 (36.1)	75 (35.2)	83 (37.0)	0.323
High triglycerides level, *n* (%)	428 (97.9)	208 (97.6)	220 (98.2)	0.746
High fasting serum glucose level, *n* (%)	207 (47.3)	99 (46.5)	108 (48.2)	0.311
High blood pressure, *n* (%)	316 (72.3)	159 (74.6)	157 (70.0)	0.336
**Medications, *n* (%)**				
Antihypertensive drugs	197 (546)	88 (41.3)	109 (48.7)	0.123
Statins	137 (31.3)	61 (28.6)	76 (33.9)	0.233
Fibrates	21 (4.8)	11 (5.2)	10 (4.5)	0.732
Hypoglycemic agents	105 (24.0)	51 (24.0)	54 (24.1)	0.365
Insulin	31 (7.1)	13 (6.1)	18 (8.0)	0.214

^a^ Blood pressure of 140/90 mm Hg or higher or use of antihypertensive drugs; ^b^ defined by a total cholesterol level of ≥200 mg/dL or use of medications for lowering the cholesterol level; ^c^ defined by a total triglyceride level of ≥150 mg/dL or use of medications for lowering the triglyceride level; ^d^ the MetS components are defined according to the harmonized criteria; the explanation of the criteria is presented in the Methods section. SD, Standard Deviation; HDL, High-Density Lipoprotein.

**Table 2:** Anthropometric and body composition variables in IGOBE program.

Variable	Global	Control Group	Intervention Group	*p*-Value
**Body weight, kg, mean (SD)**				
Basal	105.3 ± 221.5	102.6 ± 18.3	107.8 ± 23.9	0.772
1 year	102.6 ± 20.5	105.6 ± 19.4	99.7 ± 21.3	0.002
Change 1-year respect basal (%)		2.96 ± 6.13	−7.06 ± 7.26	<0.001
**BMI, kg/m^2^, mean (SD)**				
Basal	39.9 ± 7.0	39.2 ± 5.6	40.5 ± 7.9	0.716
1 year	38.8 ± 6.6	40.4 ± 6.1	37.4 ± 6.8	<0.001
Change 1-year respect basal (%)		2.90 ± 6.25	−7.33 ± 7.70	<0.001
**Waist circumference, cm, mean (SD)**				
Basal	113.9 ± 15.7	111.5 ± 15.2	115.8 ± 16.0	0.863
1 year	111.2 ± 15.3	116.5 ± 14.9	107.0 ± 14.4	<0.001
Change 1-year respect basal (%)		4.85 ± 6.43	−7.37 ± 6.9	<0.001
**Fat mass, kg, mean (SD)**				
Basal	50.2 ± 12.5	48.9 ± 11.7	51.4 ± 13.1	0.898
1 year	47.2 ± 12.3	50.3 ± 12.1	44.6 ± 11.9	<0.001
Change 1-year respect basal (%)		3.84 ± 13.77	−12.41 ± 14.8	<0.001
**Fat mass, %, mean (SD)**	48.0 ± 5.5	46.1 ± 5.8	48.3 ± 5.1	0.923
Basal	48.0 ± 5.5	46.1 ± 5.8	48.3 ± 5.1	0.923
1 year	46.3 ± 6.2	47.8 ± 5.9	44.9 ± 6.2	0.001
Change 1-year respect basal (%)		0.61 ± 10.67	−6.63 ± 11.01	<0.001
**Visceral fat, cm^2,^ mean (SD)**				
Basal	209.0 ± 50.6	201.8 ± 48.8	215.2 ± 51.6	0.914
1 year	203.5 ± 49.0	214.5 ± 49.8	194.1 ± 46.5	<0.001
Change 1-year respect basal (%)		3.84 ± 13.77	−12.41 ± 14.8	<0.001

**Table 3:** Metabolic syndrome components by group.

Variable	Control Group (*n* = 213)	Intervention Group (*n* = 224)	*p*-Value *
**MetS *n* (%)**			
Baseline prevalence	122 (57.3)	145 (64.7)	0.542
1-year prevalence	117 (55.2)	115 (51.3)	0.004
Change 1-year respect basal (%)	−2.1	−13.4	<0.001
**Abdominal obesity *n* (%)**			
Baseline prevalence	180 (84.5)	207 (92.4)	0.596
1-year prevalence	108 (50.7)	114 (50.9)	0.007
Change 1-year respect basal (%)	−33.8	−41.5	<0.001
**Low HDL level *n* (%)**			
Baseline prevalence	75 (35.2)	83 (37.0)	0.323
1-year prevalence	67 (31.5)	73 (32.6)	0.478
Change 1-year respect basal (%)	−3.7%	−4.4%	<0.001
**High triglycerides level *n* (%)**			
Baseline prevalence	208 (97.6)	220 (98.2)	0.746
1-year prevalence	196 (92.0)	216 (96.4)	0.063
Change 1-year respect basal (%)	−5.6	−1.8	<0.001
**High fasting serum glucose level or** **use of antidiabetic drugs, *n* (%)**			
Baseline prevalence	99 (46.5)	108 (48.2)	0.311
1-year prevalence	88 (41.3)	79 (35.2)	0.163
Change 1-year respect basal (%)	−5.2	−13.0	<0.001
**High blood pressure or use of antihypertensive drugs, *n* (%)**			
Baseline prevalence	159 (74.6)	157 (70.0)	0.336
1-year prevalence	145 (68.1)	139 (62.1)	0.010
Change 1-year respect basal (%)	−6.5	−7.9	<0.001

* Statistically significant interaction between groups.

**Table 4:** Medication: basal and at 12 months.

Medications, *n* (%)	Control Group (*n* = 213)	Intervention Group (*n* = 224)	*p*-Value
**Antihypertensive drugs**			
Basal	88 (41.3)	109 (48.7)	0.501
12 months	88 (41.3)	116 (51.7)	0.256
**Statins**			
Basal	61 (28.6)	76 (33.9)	0.233
12 months	67 (31.4)	78 (34.8)	0.555
**Fibrates**			
Basal	11 (5.2)	10 (4.5)	0.732
12 months	13 (6.1)	9 (4.0)	0.739
**Hypoglycemic agents**			
Basal	51 (24.0)	54 (24.1)	0.365
12 months	56 (26.3)	54 (24.1)	0.475
**Insulin**			
Basal	13 (6.1)	18 (8.0)	0.214
12 months	14 (6.5)	19 (8.5)	0.271

**Table 5:** Laboratory test results.

Variable	Control Group		Intervention Group		*p*-Value	
	Basal	1 Year	Basal	1 Year	Basal	1 Year
Fasting blood glucose mg/dL	105 ± 29	106 ± 31	107 ± 28	104 ± 30	0.851	0.494
HbA1c %	6.5 ± 1.3	6.3 ± 1.1	6.5 ± 1.3	6.1 ± 0.9	0.869	0.227
Total cholesterol, mg/dL	195 ± 38	192 ± 36	190 ± 33	191 ± 37	0.129	0.754
HDL levels, mg/dL	52 ± 14	50 ± 10	52 ± 13	52 ± 13	0.955	0.087
LDL levels, mg/dL	117 ± 32	114 ± 32	111 ± 30	112 ± 31	0.067	0.599
Triglyceride levels, mg/dL	140 ± 76	144 ± 75	133 ± 58	135 ± 62	0.304	0.177

## Data Availability

Data are available upon reasonable request from the corresponding author.
